# Diagnosing colorectal cancer in primary care: cohort study in Sweden of qualitative faecal immunochemical tests, haemoglobin levels, and platelet counts

**DOI:** 10.3399/bjgp20X713465

**Published:** 2020-11-03

**Authors:** Cecilia Högberg, Ulf Gunnarsson, Stefan Jansson, Hans Thulesius, Olof Cronberg, Mikael Lilja

**Affiliations:** Department of Public Health and Clinical Medicine;; Department of Surgical and Perioperative Sciences, Umeå University, Umeå.; University Health Care Research Centre, Örebro University, Örebro.; Department of Clinical Sciences, Lund University, Malmö; professor of primary care, Department of Medicine and Optometry, Linnaeus University, Kalmar.; Department of Clinical Sciences, Lund University, Malmö.; Department of Public Health and Clinical Medicine;

**Keywords:** anaemia, colorectal neoplasms, general practice, occult blood, thrombocytosis

## Abstract

**Background:**

Colorectal cancer (CRC) diagnostics are challenging in primary care and reliable diagnostic aids are desired. Qualitative faecal immunochemical tests (FITs) have been used for suspected CRC in Sweden since the mid-2000s, but evidence regarding their effectiveness is scarce. Anaemia and thrombocytosis are both associated with CRC.

**Aim:**

To evaluate the usefulness of qualitative FITs requested for symptomatic patients in primary care, alone and combined with findings of anaemia and thrombocytosis, in the diagnosis of CRC.

**Design and setting:**

A population-based cohort study using electronic health records and data from the Swedish Cancer Register, covering five Swedish regions.

**Method:**

Patients aged ≥18 years in the five regions who had provided FITs requested by primary care practitioners from 1 January 2015 to 31 December 2015 were identified. FIT and blood-count data were registered and all CRC diagnoses made within 2 years were retrieved. Diagnostic measurements were calculated.

**Results:**

In total, 15 789 patients provided FITs (four different brands); of these patients, 304 were later diagnosed with CRC. Haemoglobin levels were available for 13 863 patients, and platelet counts for 10 973 patients. Calculated for the different FIT brands only, the sensitivities for CRC were 81.6%–100%; specificities 65.7%–79.5%; positive predictive values 4.7%–8.1%; and negative predictive values 99.5%–100%. Calculated for the finding of either a positive FIT or anaemia, the sensitivities increased to 88.9–100%. Adding thrombocytosis did not further increase the diagnostic performance.

**Conclusion:**

Qualitative FITs requested in primary care seem to be useful as rule-in tests for referral when CRC is suspected. A negative FIT and no anaemia indicate a low risk of CRC.

## INTRODUCTION

Colorectal cancer (CRC) diagnostics are challenging, especially in a primary care context in which patients consult for a plethora of symptoms that may be associated with CRC. Rectal bleeding and change in bowel habits are generally considered as alarm symptoms, and guidelines in several countries recommend referring patients with these symptoms to secondary care, where a colonoscopy is commonly performed.^1^^–^^4^ However, the majority of patients with CRC who initially consult primary care practitioners have no alarm symptom, and alarm symptoms are also common in patients who do not have any serious diseases.^5^^–^^7^ Colonoscopies are resource heavy, uncomfortable for patients, and associated with a non-negligible risk.^8^ Reliable laboratory tests that could guide decision making on referral are desired.

In the UK, quantitative faecal immunochemical tests (FITs) for haemoglobin have been recommended since 2017 for use in primary care when CRC is suspected without the presence of alarm symptoms.^9^ These tests, which require laboratory equipment for analysis, provide a numerical value of the faecal haemoglobin concentration; the cut-off for a positive result can be set at a selected level.

In Sweden, faecal occult blood tests have been used as diagnostic tools in symptomatic patients in primary and secondary care for many years. In the mid-2000s, guaiac-based tests were replaced by qualitative FITs. These tests use a chromatographic technique in dipsticks or cassettes, with in-built, pre-set cut-offs. They are visually interpreted by identifying coloured lines, give a positive/negative result, and can easily be used as point-of-care tests. Most FITs are requested at a primary care centre and analysed there by laboratory staff; however, qualitative FITs are also used in hospitals and analysed in hospital laboratories. To the authors’ knowledge, quantitative FITs are not used anywhere in Sweden for diagnostic purposes. There is no previous or current nationwide screening programme in Sweden; one region (not included in the study presented here) started screening gradually from 2008 and some regions still plan to start screening in 2020. In spite of their frequent use, there is little evidence supporting the use of qualitative FITs as diagnostic aids.^10^^–^^12^ There are also few studies of quantitative FITs used in primary care before a referral decision is made.^13^^,^^14^

For a test to be useful in primary care, it should, ideally, have:

- high sensitivity (few false negatives) to avoid missing CRCs;
- high specificity (few false positives) to avoid unnecessary referrals;
- high positive predictive value (PPV) (a high percentage of those who test positive have CRC) to avoid unnecessary colonoscopies; and
- high negative predictive value (NPV) (a low percentage of those who test negative have CRC) to safely rule out CRC.

**Table:** How this fits in

Colorectal cancer (CRC) diagnostics are challenging in primary care and there is a desire for reliable diagnostic aids. This population-based cohort study of 15 789 patients aged ≥18 years showed that four brands of qualitative faecal immunochemical tests (FITs) requested by primary care physicians in symptomatic patients had sensitivities of 81.6%–100%, positive predictive values of 4.7%–8.1%, and negative predictive values of 99.5%–100% for CRC. The findings of either a positive FIT or anaemia increased sensitivities to 88.9%–100%. FITs seem to be useful as rule-in tests for further investigation, whereas a negative FIT and no anaemia yielded a low risk of CRC.

With serious diseases, such as CRC, it is important not to miss any cases. Combining different tests could, potentially, make it possible to attain a higher sensitivity and NPV; however, this would likely be at the expense of a lower specificity and PPV.

Anaemia has a well-known association with CRC. Not only can loss of blood result in iron deficiency and microcytic anaemia, but normocytic anaemia can also be connected to CRC.^15^^,^^16^ Anaemia is included in existing guidelines as a reason for referral.^1^^–^^3^ Thrombocytosis is connected to several forms of cancer and is associated with CRC.^17^^–^^19^ The UK’s guideline on suspected cancer features thrombocytosis as a reason for further investigation in recommendations concerning lung, upper gastrointestinal tract, and endometrial cancers.^1^

The aim of this study was to evaluate the usefulness of qualitative FITs requested by primary care practitioners, alone and in combination with findings of anaemia and thrombocytosis, in the diagnosis of CRC.

## METHOD

Patients aged ≥18 years, for whom FITs had been requested and test results had been registered in primary care between 1 January 2015 and 31 December 2015, were identified in the Swedish regions of Jämtland Härjedalen, Kronoberg, Västerbotten, Västernorrland, and Örebro. The regions were selected to represent different parts of Sweden including densely and sparsely populated areas. The total population, derived using data from Statistiska Centralbyrån (Statistics Sweden), was 1 116 751. Each region’s electronic health record (EHR) system, shared by that region’s primary care centres, was used to identify the patients. All primary care centres were included, except for four in Västerbotten (16 048 listed patients), which had separate health-record systems. All FIT registration dates and results were recorded.

The authors considered faecal samples registered within 14 days as belonging to the same FIT, and the date of each FIT was set at the date of the first sample. The FITs were analysed by laboratory staff at each primary care centre (supervised by each region’s hospital laboratories) or sent to the hospital laboratories. Instructions on which FIT brand to use, sampling, storage, and analysis were given by each region’s central hospital laboratory (in accordance with manufacturers’ instructions) and followed by all primary care centres and hospital laboratories in that region. If ≥1 of the samples showed a positive result, the FIT was considered to be positive; if >1 FIT was carried out during 2015, the first FIT was included in the analysis. In Sweden, it is customary to request three samples for one FIT, with samples collected from consecutive bowel movements but not >1 sample per day. The analysis focused on those patients who had provided FITs with exactly three samples (three-sample FITs).

Four brands of visually read qualitative FITs were used:

- Actim Fecal Blood (Oy Medix Biochemica AB, Finland) in Örebro;
- Analyz FOB (LumiraDx AB, Sweden) in Kronoberg, Västerbotten, and Västernorrland;
- Chemtrue FOB Test (Chemtron Biotech Co Ltd, China) in Jämtland Härjedalen; and
- Diaquick FOB (Dialab GmbH, Austria) in Kronoberg.

According to the manufacturers, at the time of the study the test properties were as follows: Actim Fecal Blood used a dipstick device for analysis, whereas the others used cassettes with sample wells. A positive test was identified with the visual reading of a coloured line. In 2015, Actim Fecal Blood had a cut-off level of 25–50 µg/g faeces; Analyz FOB had a cut-off level of 2 µg/g faeces; Chemtrue FOB Test used 40 ng/ml faecal solution (data on cut-off level in µg/g not available); and Diaquick FOB had a cut-off level of 5 µg/g faeces. They all showed positive results up to, at least, haemoglobin concentrations of 0.5 mg/ml. All devices featured a built-in control line to indicate proper performance of the test and the device. Once collected, Actim Fecal Blood could be stored at room temperature for 7 days before analysis; Analyz FOB and the Chemtrue FOB Test could be stored for 15 days; and Diaquick FOB could be stored for 3 days.

Data on haemoglobin levels and platelet counts were collected from the same EHRs from 1 month before until 1 month after the date of each FIT. These tests were analysed at the regions’ hospital laboratories or the primary care centres’ laboratories, all of which were accredited by Swedac (Sweden’s national accreditation body). If >1 test had been analysed, the test result closest to the FIT date was registered.

Anaemia and thrombocytosis were defined in line with the reference standards used by the laboratories; they, in turn, base these on results from the Nordic Reference Interval Project. Anaemia was defined as:

- haemoglobin level: <117 g/l in females in all regions; and
- haemoglobin level: <134 g/l in males in all regions.

Thrombocytosis was defined as:

- platelet count: >390 × 10^9^/l in females and >350 × 10^9^/l in males in Örebro; and
- platelet count: >387 × 10^9^/l in females and >348 × 10^9^/l in males in the other regions.

Information about patients diagnosed with CRC within 2 years of the FITs was obtained from the Swedish Cancer Register. The limit of 2 years was chosen as this is the recommended screening interval time in Europe.^20^ It has also been used in prior studies undertaken in primary care, such as those by Högberg *et al* ^10^^,^^11^ and Mowat *et al*.^14^ No patients included in the study presented here had participated in any ongoing or previous screening for CRC as there had been no national, regional, or local screening for CRC in the areas, and there was no national standardised care pathway concerning CRC at the time of the study. The study followed the Standards for Reporting of Diagnostic Accuracy Studies 2015 guidelines.^21^

### Sample size

As the study only includes diagnostic measurements and no hypothesis testing was planned, no power calculation is presented. Instead, when calculating the probability of a test showing no CRC, estimated confidence intervals (CIs) were used and the probability that half of the CI would, at the most, reach a specified value. With an assumed CI of 0.99 (standard deviation 0.002), this probability would be >80% with a total number of 10 283 patients.

### Statistics

IBM SPSS Statistics (version 24) was used for statistical analyses. Sensitivities, specificities, PPVs, and NPVs with 95% CIs and likelihood ratios (LRs) were calculated for FIT results, both alone and combined with findings of anaemia and thrombocytosis, for the diagnosis of CRC.

## RESULTS

**Figure 1.**
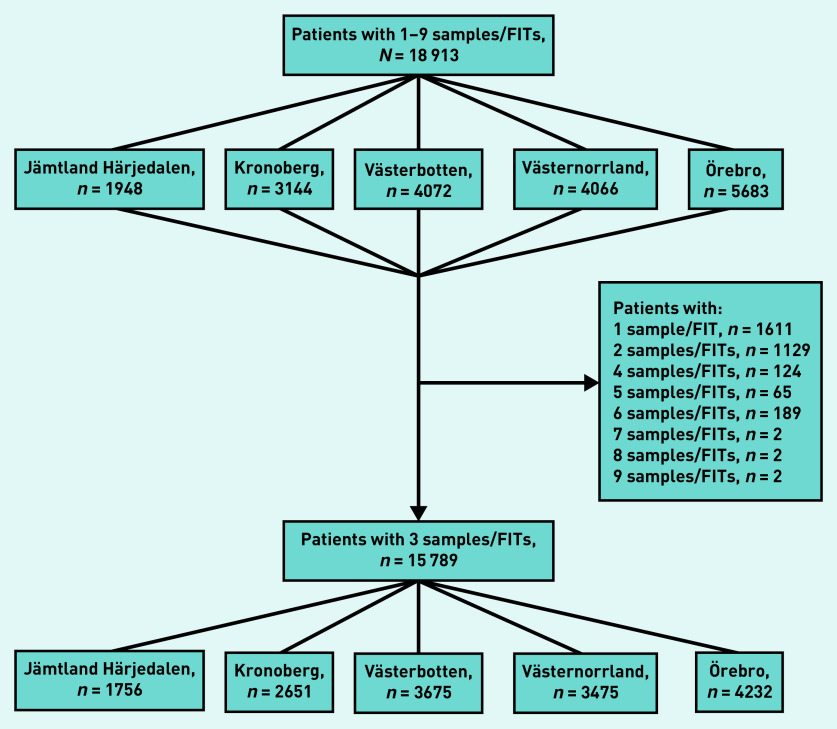
**Patient locations and number of samples. FIT = faecal immunochemical test.**

FITs with between one and nine samples were provided by 18 913 patients, 60.4% of whom were female. Patients’ median age was 65 (interquartile range [IQR] 48–75) years. Of this group, 15 789 (60.9% female; median age 65 [IQR 47–75] years) provided three-sample FITs (Figure 1); 29.0% of these showed a positive result. The demographic characteristics of this patient sample are outlined in Table 1.

**Table 1.** Demographic characteristics of symptomatic patients who provided three-sample FITs in primary care from 1 January 2015 to 31 December 2015,^a^ stratified by region

**Characteristic**	**Region**					**Total**

	**Jämtland Härjedalen**	**Kronoberg**	**Västerbotten**	**Västernorrland**	**Örebro**	
Population, *n*	127 169	191 062	263 584	244 046	290 890	1 116 751

Patients, *n* (%)	1756 (1.38)	2651 (1.39)	3675 (1.39)	3475 (1.42)	4232 (1.45)	15 789 (1.41)

Sex, female, %	62.2	59.4	60.8	61.6	60.7	60.9

Median age, years (IQR)	67 (51–76)	65 (49–75)	63 (46–75)	66 (50–75)	62 (43–74)	65 (47–75)

Aged ≥40 years, *n* (%)	1503 (85.6)	2276 (85.9)	3026 (82.3)	2987 (86.0)	3337 (78.9)	13 129 (83.2)

Patients diagnosed with CRC, *n*	29	50	75	71	79	304

FIT brand, *n*						
Actim Fecal Blood	—	—	—	—	4232	4232
Analyz FOB	—	739	3675	3475	—	7889
Chemtrue FOB Test	1756	—	—	—	—	1756
Diaquick FOB	—	1751	—	—	—	1751
Analyz FOB or Diaquick FOB	—	161	—	—	—	161

a Population as at 1 November 2015. CRC = colorectal cancer. FIT = faecal immunochemical test. IQR = interquartile range.

Of the three-sample FITs, 50.0% (*n* = 7889) were Analyz FOB, 26.8% (*n* = 4232) Actim Fecal Blood, 11.1% (*n* = 1756) Chemtrue FOB Test, and 11.1% (*n* = 1571) Diaquick FOB. Kronoberg changed from using Diaquick FOB to Analyz FOB during 2015 and, for 161 patients (1.0%), it was unclear which FIT had been used. Details on each FIT’s diagnostic performance are presented in Table 2. Of the 15 789 patients who provided three-sample FITs, 304 (Table 1) (1.9%; 49.0% female; median age 73 [IQR 66–80] years) were diagnosed with CRC within 2 years; 277 (1.8%) had positive FITs, and 27 (0.2%) had false negative FITs. A separate calculation for a 1-year follow-up period, instead of the 2-year period, showed 28 fewer cases of CRC, an overall sensitivity of 93.8%, specificity of 72.2%, a PPV of 5.7%, and an NPV of 99.8% (data not shown).

**Table 2.** Test results for three-sample FITs requested in primary care in symptomatic patients, stratified for different test brands and related to diagnoses of CRC

**Test result**	**All FIT brands, *n*= 15 789**	**Actim Fecal Blood, *n*= 4232**	**Analyz FOB, *n*= 7889**	**Chemtrue FOB Test, *n*= 1756**	**Diaquick FOB, *n*= 1751**	**Diaquick FOB or Analyz FOB, *n*= 161**
Colorectal cancer, *n*	304	79	158	29	38	0
True positive, *n*	277	77	140	29	31	0
False negative, *n*	27	2	18	0	7	0
False positive, *n*	4298	1230	2090	593	352	33
True negative, *n*	11 187	2923	5641	1134	1361	128
Sensitivity, %	91.1	97.5	88.6	100	81.6	n/a
Specificity, %	72.2	70.4	73.0	65.7	79.5	79.5
PPV (95% CI)	6.1 (5.4 to 6.8)	5.9 (4.6 to 7.2)	6.3 (5.3 to 7.3)	4.7 (3.0 to 6.3)	8.1 (5.4 to 10.8)	n/a
NPV (95% CI)	99.8 (99.7 to 99.8)	99.9 (99.8 to 100)	99.7 (99.5 to 99.8)	100 (99.7 to 100)	99.5 (99.1 to 99.9)	100 (97.2 to 100)
LR+	3.28	3.29	3.28	2.92	3.98	n/a
LR–	0.12	0.04	0.16	0	0.23	n/a

CRC = colorectal cancer. FIT = faecal immunochemical test. LR− = negative likelihood ratio. LR+ = positive likelihood ratio. n/a = not available. NPV = negative predictive value.

PPV = positive predictive value.

The PPVs for CRC of three-sample FITs were 4.7%–8.1% for patients of all ages; 5.3%–9.0% for those aged ≥40 years; and 5.7%–10.3% for those aged ≥60 years when calculated for each brand separately (Supplementary Table S1). Calculated for all FIT brands together, the PPV was 6.1% (95% CI = 5.4 to 6.7) when calculated for all patients aged ≥18 years; 6.8% for those aged ≥40 years (95% CI = 6.1 to 7.6); and 7.6% (95% CI = 6.7 to 8.6) for those aged ≥60 years (Table 3). There was a tendency towards lower specificities with increasing age.

**Table 3.** Test results for three-sample FITs (all FIT brands) requested in primary care in symptomatic patients, stratified by age and related to CRC diagnoses

**Test result**	**Aged ≥18 years, *n*= 15 789**	**Aged ≥40 years, *n*= 13 129**	**Aged ≥50 years, *n*= 11 375**	**Aged ≥60 years, *n*= 9266**	**Aged ≥70 years, *n*= 6034**	**Aged ≥80 years, *n*= 2328**
Colorectal cancer, *n*	304	301	290	263	196	78
True positive, *n*	277	275	265	240	176	70
False negative, *n*	27	26	25	23	20	8
False positive, *n*	4298	3746	3394	2898	2018	862
True negative, *n*	11 187	9082	7691	6105	3820	1388
Sensitivity, %	91.1	91.4	91.4	91.3	89.8	89.7
Specificity, %	72.2	70.8	69.4	67.8	65.4	61.7
PPV (95% CI)	6.1 (5.4 to 6.7)	6.8 (6.1 to 7.6)	7.2 (6.4 to 8.1)	7.6 (6.7 to 8.6)	8.0 (6.9 to 9.2)	7.5 (5.8 to 9.2)
NPV (95% CI)	99.8 (99.7 to 99.8)	99.7 (99.6 to 99.8)	99.7 (99.5 to 99.8)	99.6 (99.5 to 99.8)	99.5 (99.3 to 99.7)	99.4 (99.0 to 99.8)
LR+	3.28	3.13	2.99	2.84	2.60	2.34
LR–	0.12	0.12	0.12	0.13	0.16	0.17

CI = confidence interval. CRC = colorectal cancer. FIT = faecal immunochemical test. LR− = negative likelihood ratio. LR+ = positive likelihood ratio. NPV = negative predictive value.

PPV = positive predictive value.

For patients with CRC, all three faecal samples tested positive for 228 patients; two out of three samples tested positive for 29 patients; and one out of three for 20 patients. Of the 27 patients with CRC who had negative FITs, 18 had tumours in the right colon, six in the left colon, and three in the rectum; nine had anaemia. The median time from the FIT to the colorectal diagnosis was 62 (IQR 35–110) days for patients with positive FITs and 185 (IQR 87–467) days for those with negative FITs (data not shown).

Haemoglobin levels were available for 13 863 (87.8%; 60.7% female; median age 65 [IQR 48–75] years) of the 15 789 patients who provided three-sample FITs; platelet counts as well as haemoglobin levels were available for 10 973 patients (69.5%; 60.5% female; median age 66 [IQR 49–76] years). The results for combinations of tests for each separate FIT brand are presented in Supplementary Tables S2–S5; results for all FIT brands combined are given in Table 4.

**Table 4.** All FIT brands: test results for combinations of three-sample FITs, haemoglobin values, and platelet counts in symptomatic patients in primary care related to diagnoses of CRC

**Test result**	**FIT and haemoglobin value, *n*= 13 863**				**FIT, haemoglobin value, and platelet count, *n*= 10 973**				
	**Positive FIT**	**Anaemia^a^**	**Anaemia^a^ and positive FIT**	**Anaemia^a^ and/or positive FIT**	**Positive FIT**	**Thrombocytosis^b^**	**Thrombocytosis^b^ and positive FIT**	**Thrombocytosis^b^ and/or positive FIT**	**Thrombocytosis^b^ and/or anaemia^a^ and/or positive FIT**
Colorectal cancer, *n*	289	289	289	289	237	237	237	237	237
True positive, *n*	262	132	123	271	212	40	39	213	222
False negative, *n*	27	157	166	18	25	197	198	24	15
False positive, *n*	3858	3685	1356	6187	3135	875	355	3655	5394
True negative, *n*	9716	9889	12 218	7387	7601	9861	10 381	7081	5342
Sensitivity, %	90.7	45.7	42.6	93.8	89.5	16.9	16.5	89.9	93.7
Specificity, %	71.6	72.9	90.0	54.4	70.8	91.8	96.7	66.0	49.8
PPV (95% CI)	6.4.	3.5 (2.9 to 4.1)	8.3 (6.9 to 9.7)	4.2 (3.7 to 4.7)	6.3 (5.5 to 7.2)	4.4 (3.0 to 5.7)	9.9 (6.9 to 12.8)	5.5 (4.8 to 6.2)	4.0 (3.5 to 4.5)
NPV (95% CI)	99.7 (99.6 to 99.8)	98.4 (98.2 to 98.7)	98.7 (98.5 to 98.9)	99.8 (99.6 to 99.9)	99.7 (99.5 to 99.8)	98.0 (97.8 to 98.3)	98.1 (97.9 to 98.4)	99.7 (99.5 to 99.8)	99.7 (99.6 to 99.9)
LR+	3.19	1.69	4.26	2.06	3.07	2.06	5.00	2.64	1.87
LR–	0.13	0.74	0.64	0.11	0.15	0.91	0.86	0.15	0.13

a Anaemia: haemoglobin value< 117 g/l in females, < 134 g/l in males.

b Thrombocytosis: > 387 × 10^9^/l in females and > 348 × 10^9^/l in males in Jämtland Härjedalen, Kronoberg, Västernorrland, Västerbotten; > 390 × 10^9^/l in females and >350x10^9^/l in males in Örebro. CI = confidence interval. CRC = colorectal cancer. FIT = faecal immunochemical test. LR− = negative likelihood ratio. LR+ = positive likelihood ratio. NPV = negative predictive value. PPV = positive predictive value.

Of the 40 patients with CRC and thrombocytosis, one patient (with platelet count of 376 × 10^9^/L) had a negative FIT and 29 had both anaemia and thrombocytosis (data not shown). Calculated for all FIT brands and patients aged ≥40 years, the PPV for CRC of anaemia was 3.8%; of anaemia and positive FIT 8.7%; of either anaemia or positive FIT 4.7%; of thrombocytosis 4.8%; of thrombocytosis and positive FIT 10.7%; and of either thrombocytosis or anaemia or positive FIT 4.4% (data not shown). Sensitivities were the same as when calculated for all ages. When assessing thrombocytosis for CRC, the use of a higher threshold to define thrombocytosis — namely, a platelet count of >400 × 10^9^/l for both sexes — resulted in a PPV of 3.5% (95% CI = 1.8 to 5.1) for females and 6.1% (95% CI = 2.0 to 10.1) for males when calculated for all ages (data not shown).

## DISCUSSION

### Summary

This population-based cohort study of 15 789 patients with four brands of qualitative three-sample FITs, requested by primary care practitioners as diagnostic aids, showed sensitivities of 81.6%–100%, specificities of 65.7%–79.5%, PPVs of 4.7%–8.1%, and NPVs of 99.5%–100% for CRC. CRC was diagnosed in 304 patients within 2 years of the FIT having been undertaken. Calculated for the finding of either a positive FIT or anaemia, the sensitivities improved to 88.9%–100%. Adding thrombocytosis did not further increase the overall diagnostic performance, although one in 10 patients with a positive FIT and thrombocytosis were diagnosed as having CRC.

### Strengths and limitations

This study has several strengths. It includes a large, population-based number of patients from both cities and sparsely populated areas in Sweden. Data were collected from EHRs with a complete coverage, except for a small number of inhabitants, as described in the Background section. It is highly probable that the vast majority of cases of CRC were retrieved, as the Swedish Cancer Register has a completeness of almost 100%.^22^

Four different brands of FIT were used, with cut-offs of 2–50 µgHb/g faeces, in line with manufacturers’ instructions. Potentially, the different cut-offs should have been reflected in higher sensitivities for brands with lower cut-offs, but this did not seem to be the case. For example, Actim Fecal Blood (stated cut-off of 25–50 µg/g faeces) showed a higher sensitivity of 97.5% for CRC than Diaquick FOB (stated cut-off of 5 µg/g faeces), which had a sensitivity of 81.6%. Differences in age distribution between the regions may have influenced the calculated sensitivities; however, this variation between brands has also been described previously for qualitative, as well as quantitative, FITs.^23^^,^^24^ Despite the variation in stated cut-offs, all brands had sensitivities of >80%. With a focus on one brand only it would have been easier to interpret the results; however, having examined several, the findings showed that brands with different stated cut-offs do not necessarily show corresponding differences in sensitivities. It also reflects the clinical situation, where different brands are used.

The authors do not know which symptoms prompted the primary care practitioners to request FITs, but the results, presumably, reflect the clinical situations and the primary care practitioners’ practices. To the authors’ knowledge, only one prospective study has examined which symptoms Swedish primary care practitioners register when FITs are requested; this showed abdominal pain (57%), change in bowel habits (44%), diarrhoea (43%), rectal bleeding (25%), urgency (20%), and anaemia (17%) to be the main symptoms.^11^ The organisation of the Swedish healthcare system is uniform and it seems probable that the primary care practitioners in the study presented here requested FITs for similar reasons. There might have been differences between the regions in how generous primary care practitioners were with FIT requests, which could affect the PPVs and NPVs; however, the percentage of inhabitants that provided three-sample FITs was of similar magnitude (1.38%–1.45%) in all of the regions included.

The FITs were analysed by many different people at primary care centres and hospital laboratories, which may have resulted in a variation in the interpretation of test results; however, all primary care centre and hospital laboratory staff were supervised by the central laboratory in each region. In addition, there was an in-built control line in each FIT device.

This study was limited to CRC as it was not possible to find reliable information about other diagnoses. If, for example, adenomas with high-grade dysplasia and inflammatory bowel diseases had been included, the PPVs for FITs would likely have been higher.^11^^,^^25^

### Comparison with existing literature

To the authors’ knowledge, this is the first population-based cohort study on qualitative FITs requested for symptomatic patients in primary care before referral, the first to include >1 qualitative FIT brand, and the first to study FITs in combination with haemoglobin levels and platelet counts.

The use of different FIT brands, age limits, and follow-up periods complicates study comparisons. The incidence of CRC increases with age, and the PPVs of FITs for CRC were shown to be higher for older age groups. The authors became aware of two studies with similar findings to those presented here that have reported on the use of a qualitative FIT brand (Actim Fecal Blood) on symptomatic patients in primary care before referral: one retrospective (age ≥18 years, follow-up 2 years) and one prospective (age ≥20 years, follow-up 2 years).^10^^,^^11^ These studies reported sensitivities of 88% and 87.5%, specificities of 74% and 67.4%, PPVs of 6.7% and 5.6%, and NPVs of 99.7% and 99.6%, respectively, for FITs for CRC.

The authors also found two studies (one Danish,^13^ one Scottish;^14^ both prospective) reporting on the use of quantitative FITs with cut-offs of ≥10 µgHb/g faeces for symptomatic patients before referral. The Danish study^13^ included patients without alarm symptoms (age ≥30 years) and showed a PPV for CRC of 9.4%, which is higher than found in this study. A lower FIT positivity rate of 15.6% was identified (compared with 29.0% in this study), which could indicate differences in the FITs’ sensitivities; however, this is hard to determine, as CRCs with negative FITs may have been missed with only 3-month follow-up in the Danish study. The Scottish study^14^ including all ages (follow-up 2 years) with a FIT positivity rate of 21.9% did not present any sensitivity or PPV, but concluded that FITs combined with clinical assessment could safely determine a patient’s risk of CRC.

Further studies have reported on the use of quantitative FITs on symptomatic patients who have already been referred. The authors found five studies^25^^–^^29^ indicating similar CRC prevalence (2.1%–5.2%) as in the present study, with patients aged ≥16 years or ≥18 years who underwent endoscopy; one-sample FITs with cut-offs of 10–15 µg/g faeces were used in these studies and they presented sensitivities of 84.6%–100%, specificities of 76.5%–93.9%, and PPVs of 7.6%–28.6%.

Combining the findings of a positive FIT or anaemia increased the sensitivity for CRC; this has also been observed in other studies, one in primary care in Sweden and one concerning patients referred on the 2-week-wait pathway in England.^11^^,^^30^

Thrombocytosis has been shown to be associated with cancer diagnoses, especially CRC and lung cancer.^17^^–^^19^ In the present study, one in 10 patients with thrombocytosis combined with a positive FIT was diagnosed as having CRC; however, all patients with CRC and thrombocytosis, except one, also had a positive FIT. The sensitivity of thrombocytosis alone for CRC was found to be low, making it unsuitable as a single diagnostic test for CRC. Yet the present study confirms the connection between high platelet counts and cancer in primary care patients.^18^

UK guidance from the National Institute for Health and Care Excellence on suspected cancer recommends a PPV threshold of 3%, above which investigation or referral is warranted;^1^ the PPV of a positive FIT in this study is well above that threshold. The finding of either a positive FIT or anaemia, with PPVs of 3.8 to 5.0 for the different FIT brands, also appears to be useful and has the advantage of a higher sensitivity. Adding platelet count did not further increase the sensitivity for CRC. The authors have found no other studies reporting on the use of FITs combined with haemoglobin levels and platelet counts for comparison.

### Implications for research and practice

Qualitative FITs seem to be useful as rule-in tests in primary care to select patients for investigation of suspected CRC. For better sensitivity, it seems advantageous to combine FITs with the assessment of haemoglobin levels. The combination of a negative FIT and no anaemia indicates a low risk of CRC in this study — a combination that could potentially be helpful as a rule-out test. Both FITs and blood counts are easy to carry out at low cost; however, further prospective studies are needed to confirm the findings presented here.

As this Swedish study was performed before the introduction of standardised care pathways for cancer, an unknown proportion of patients with alarm symptoms was included. Previous studies including patients who have already been referred indicate that FITs could also be useful when selecting patients for bowel investigation who have histories of rectal bleeding or a change in bowel habits.^31^^–^^33^ Further studies in primary care are needed to evaluate whether there are, ultimately, any symptoms indicating when FITs should or should not be used.

Three-sample FITs were used in this study; had one-sample FITs been used, the sensitivity would have likely been lower.^34^ One-sample FITs with a verified low cut-off could, perhaps, provide sufficient sensitivity; however, CRCs can bleed intermittently and samples from >1 day are more likely to detect CRC. Further studies are needed to determine the optimal cut-off and number of samples.

There was a variability in sensitivity between the FIT brands included in this study that did not correspond to the cut-off values provided by the manufacturers; health authorities should be aware of this when deciding on which brand to use. For diagnostic purposes in patients who are symptomatic, a high sensitivity and NPV is important. Also, there is a need to standardise FIT methods, so different brands can be more easily compared.

The time interval from the FIT being undertaken to a CRC diagnosis being made was longer for patients with negative FITs. It is important for primary care practitioners to be aware that FITs do not identify all CRCs; however, FITs seem to be useful as rule-in tests for referral, and a negative FIT combined with no anaemia yields a low risk of CRC.
